# When Punishment Pays

**DOI:** 10.1371/journal.pone.0057378

**Published:** 2013-03-06

**Authors:** Gilbert Roberts

**Affiliations:** Centre for Behaviour and Evolution, Institute of Neuroscience, Newcastle University, Newcastle, United Kingdom; Universidad Carlos III de Madrid, Spain

## Abstract

Explaining cooperation in groups remains a key problem because reciprocity breaks down between more than two. Punishing individuals who contribute little provides a potential answer but changes the dilemma to why pay the costs of punishing which, like cooperation itself, provides a public good. Nevertheless, people are observed to punish others in behavioural economic games, posing a problem for existing theory which highlights the difficulty in explaining the spread and persistence of punishment. Here, I consider the apparent mismatch between theory and evidence and show by means of instructive analysis and simulation how much of the experimental evidence for punishment comes from scenarios in which punishers may expect to obtain a net benefit from punishing free-riders. In repeated games within groups, punishment works by imposing costs on defectors so that it pays them to switch to cooperating. Both punishers and non-punishers then benefit from the resulting increase in cooperation, hence investing in punishment can constitute a social dilemma. However, I show the conditions in which the benefits of increased cooperation are so great that they more than offset the costs of punishing, thereby removing the temptation to free-ride on others' investments and making punishment explicable in terms of direct self-interest. Crucially, this is because of the leveraging effect imposed in typical studies whereby people can pay a small cost to inflict a heavy loss on a punished individual. In contrast to previous models suggesting punishment is disadvantaged when rare, I show it can invade until it comes into a producer-scrounger equilibrium with non-punishers. I conclude that adding punishment to an iterated public goods game can solve the problem of achieving cooperation by removing the social dilemma.

## Introduction

The social dilemma of a public goods game arises because one makes a profit on others' investments but a loss on one's own. Reciprocity has been shown to be an effective solution to the problem of cooperation in pairs [1] but breaks down in larger groups because it is not possible to retaliate against individual defectors without damaging group cooperation further [2]. How cooperation may be established in public goods dilemmas in groups of more than two remains a key problem. In principle punishment provides a way of overcoming this problem by allowing retaliation against individuals [3]. However, punishment is itself costly to perform. As such, it is widely stated that punishment poses a public goods dilemma, just as does cooperation itself and hence that both its emergence and its stability raise major questions. ‘Second order’ free-riders who fail to punish, even if they contribute to the joint enterprise, should theoretically invade punishers, leading to unstable punishment and in turn reduced cooperation [4]–[12]. Hence, far from solving the problem of explaining cooperation in groups, punishment seems simply to replace it with a new conundrum [13].

Nevertheless, punishment is widespread in human societies and also occurs in other species [14]. Examples include punishing subordinates for failing to help in superb fairy wrens [15]; chasing cleaner-fish that feed on host tissue rather than ectoparasites [16]; and punishing subordinates for pregnancy in meerkats [17]. A particular focus has been on studying punishment in humans in the context of experimental economic games [18]–[24]. Typically, the design of such experiments is to invite participants to play public goods games which are followed by an opportunity to punish others in the light of their contributions. Punishment typically costs the punisher and inflicts a greater cost on the punished. Studies have used either a “stranger” design in which group membership changes on each round [18] or a “partner” design in which individuals interact repeatedly within the same group [19]–[22], [25]. In both designs, the fact that people will punish others at a cost to themselves has been considered “altruistic” [18] leading to the suggestion that it might be explained by group-selected other-regarding preferences [26]. However, punishment in the Fehr & Gachter experiment reduced both individual and group payoffs, so groups with punishment would actually do worse than those without. In contrast, more recent work using a partner design with larger numbers of rounds has demonstrated that punishment can increase group payoffs. Nevertheless, this has been interpreted as evidence for group selection in the sense of individuals paying a cost to benefit the group [20].

A number of theoretical models have attempted to explain punishment. One suggestion is that when defectors are rare, the costs of punishment become low so it may be maintained in the population [6], [27]. However, these models cannot account for the initial establishment of cooperation and punishment. Nor can the group level benefits upon which such models are based explain why individuals punish even when this reduces group payoffs as in [18]. A recent model suggests punishment can spread when it is coordinated [12], yet this argument appears to suffer from the logical flaw that if everyone can agree to punish, then everyone could agree to cooperate in the first place, making punishment redundant. Another idea is that the ability to opt-out of participation in public goods production and use can facilitate the spread of punishment [10], [28], but this depends on restrictive assumptions [29]. Alternatively, allowing individuals to develop a reputation for punishing may enhance the spread of punishment and thereby cooperation [22], [30], [31]. Recent models conclude that while punishment might in principle be selfish, selection is unlikely to support punishment under any “interesting” conditions [32], [33]. In consequence, a review concluded that explaining the observed tendency of people to punish “remains an open issue” [4].

There are two issues here: most crucially, why do people punish at all when it is costly, and secondly why does this continue when people are told they are anonymous and will only meet once. The latter issue has been addressed in general terms by a number of authors [34]–[37]. A full discussion is beyond the scope of this article, but here I take the view that natural selection has fashioned psychological mechanisms which lead us to behave in ways which are on average adaptive. Therefore any particular instance of a behaviour such as cooperation or punishment needs to be understood in the context of us having, for example, reward centres that are activated by cooperating or punishing [38], [39]. The key question is the functional one of why punishing is in general adaptive so that we are designed with reward systems that make punishment an essentially emotional response [39]. If we can explain that, then we can postulate why we have the psychological mechanisms that motivate us to punish even when in the somewhat abstract confines of a one-shot economic experimental game this may not always be in our best monetary interests (e.g. [40] p. 126; [41]). This logic holds for punishment just as much as for cooperation. It is typically found that people cooperate more than predicted in one-shot encounters [42]. Yet this can be understood in terms of the benefits of cooperating in repeated encounters [1] making it worth having a disposition towards being ‘nice’, i.e. commencing an interaction with cooperation [43]. Similarly, I consider the key question to be why should people be designed with a propensity to punish? To answer this we first need to understand under what conditions punishment might on average be in one's own self interest. Just as with cooperation, the question becomes, when can it be worth paying a short term cost in order to on average receive more substantial long term benefits? Such an approach acknowledges that cultural processes as well as genetic evolution may have played an important role in determining human punishment norms [23], [44]. My focus on repeated games reflects the fact that these are the most relevant for understanding how cooperation has evolved in human groups, and is consistent with the focus of much recent work on punishment [20], [21], [45], [46]. Some authors have of course already pointed out that for punishment to evolve it must have some direct or indirect benefits [3], [6], [47], [48]. Here I elucidate the way in which such direct benefits may arise from punishment and thereby resolve the second order dilemma in repeated games. In doing so I focus on the extent to which such benefits might explain recent evidence for punishment in behavioural economic games.

## Analysis of when punishment pays in repeated games

Here I develop an analytical treatment of when punishment pays in repeated games. It is almost universally assumed that costly behaviours pose a social dilemma e.g. [4], [12], [18], [20]. Of course, such a dilemma may be resolved through reciprocity, but it is rarely considered that behaviours which benefit others may also bring direct fitness benefits to the actor which offset their short term cost without any need for reciprocity [49]. I show here that within a wide parameter space typical of experimental economic games, punishment is self-interested and that it therefore does not pose a second order dilemma. The superimposition of this self-interested punishment game on top of a public goods dilemma can solve the problem of cooperation in groups and explain why people are observed to punish others.

To see how punishment can be self-interested, consider how punishing works through increasing the payoff for cooperation relative to defection. Here I consider repeated games between the same sets of players in which players first decide whether to cooperate or defect and secondly make a decision about whether to punish defectors. I consider decisions about whether or not to cooperate and whether or not to punish as being distinct. This avoids the linkage between the traits typically assumed in previous treatments in which cooperators punish [3], [33] and allows these two domains of prosociality to evolve independently. Assuming that individuals choose between cooperating and defecting according to which generates the higher payoff, then a defector should respond to being punished by switching to cooperating when the payoffs for defecting decline below the payoffs for cooperating, i.e. when:
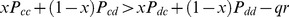
where Pcd is the payoff to a cooperator interacting with defector, co-operators are in proportion x, q is the cost incurred on being punished and r is the average rate of punishment experienced by a defector. If cooperation has net cost c to the cooperator and benefit b to all other group members, this becomes:
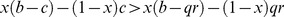
which simplifies to
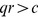
(1)This means that defectors should switch to cooperating when the costs they incur by being punished exceed the net costs of cooperating (cf. [6]). I refer to such defectors as ‘responsive defectors’.

Note that the analysis here is based on the rationale that punishment is a means by which defectors can be encouraged to cooperate. If co-operators are punished then cooperation is always uneconomic, since they bear both the costs of cooperating and the costs of being punished whereas defectors bear neither. The phenomenon of antisocial punishment [24] cannot therefore be explained as a means of encouraging cooperation.

Secondly consider how the payoffs to co-operators vary with the frequency of co-operators. As above, the payoff *P* to co-operators at frequency *x* is
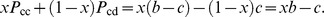
Thus, the payoff to cooperators increases with their frequency. So by punishing responsive defectors and thereby encouraging them to switch to cooperation, punishers could in principle benefit themselves. The problem is that non-punishers also benefit from any increase in cooperators. It therefore appears that punishers should do less well than others who do not punish. So why pay the cost of punishing?

Following (Roberts 2005) consider the scenario in Table 1a in which a punisher and a non-punisher are in a group with a responsive defector. Here *p* is the cost of carrying out one unit of punishment and s is an individual's benefits arising from the expected increase in cooperation when any group member punishes. Provided *s*>*p* then there is no temptation to avoid paying the costs of punishing: punishment is always the best option so will be favoured by individual self-interest. In other words, provided the gains from increased cooperativeness exceed the costs of punishing, punishing can be self-interested. In this case punishment is an example of “weak altruism” ([50] see [49] and [36] for analysis of such instances).

**Table 1:** Whether or not to punish a defector.

a		
	Punisher	Non-punisher
Punisher	2*s* - *p*	*s* - *p*
Non-punisher	*s*	0

b		
	Punisher	Non-punisher
Punisher	*s* - *p*	*s* - *p*
Non-punisher	*s*	0

Payoffs to punishers and non-punishers when with a defector in a group of three. Payoffs are shown for the row player only. *p* is the cost of carrying out one unit of punishment and *s* is the benefit an individual receives when any group member punishes. This benefit is assumed to arise from defectors switching to cooperating after being punished. Provided *s>p* punishment pays. In (a) each act of punishment has an additive effect on the benefits arising, whereas in (b) punishing when another individual is already punishing brings no additional benefit.

How can we calculate the benefits of punishing denoted by *s* above? Following on from the derivation in Equation 1 above that responsive defectors should switch to cooperating when the costs of being punished exceed those invested, in effect every unit lost to being punished means an additional unit should be invested in cooperating. The additional benefits arising to a punisher as a result of an act of punishment will therefore be the extra number of acts of cooperation performed over the remaining rounds (which is simply the cost incurred on being punished *q* divided by the net cost of cooperating *c*) multiplied by the extra benefits *b* to the individual of each cooperation. Thus the benefits of punishing are:
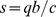
and the condition for punishment to pay is where these benefits exceeds the costs of punishment *p*, i.e where:
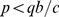
(2)In other words, the act of punishing (carried out by a punisher who may be either a cooperator or a defector) will invade when its effect on switching a defector to cooperating brings a net return to the punisher through increased cooperation. For simplicity, this assumes both that there are sufficient remaining rounds for the additional cooperative acts to take place and that cooperation is not already at a maximal level.

Note that an investment *i* results in benefit *ki*/*g* where *k* is the multiple applied to cooperative investments when pooled and *g* is group size. The net cost *c* = *i*-*ki*/*g*. In typical experiments [18], [20], [23] parameters are *k* = 2, *g* = 3, *p* = 1, *q* = 3 (or similar) and so for *i* = 1 we have *c* = 0.33 and *b* = 0.67. Solving the punishment condition we can see that for every unit invested in punishment, the punisher obtains 6 units in return. A social dilemma only exists where the return on investment is less than 1 (e.g. if contributions to a group pool are doubled and then divided by three group members then each unit invested only yields 0.67 in return). Therefore with these parameters punishment does not pose a social dilemma and is in fact in the direct self-interest of the punisher.

The above punishment ratio of 1∶3 is widely used without justification being offered. Whereas in laboratory economic games one can set sanctioning regimes arbitrarily, the question arises as to whether effective punishment of free-riders could be achieved in more real-world settings with such high relative costs to the punished. One possibility favouring punishment is that there are strong asymmetries between dominant punishers and subordinates that are punished [14]. However, if interactions are symmetric, punished individuals may equally well retaliate. Such retaliation may be incorporated most simply by considering *p* and *q* to be the net costs following a cycle of punishment with retaliation, which we would expect to give a ration of equivalence. It is interesting to note that with *p* = 1, *q* = 1 and keeping *k* = 2, *g* = 3 then investment in punishment still reaps double the rewards from increased cooperation. This counterintuitive result is due to the fact that costs inflicted by punishment translate into net costs of investing in cooperation; the actual investment in cooperation is then *i* = *c*/(1−*k*/*g*) = 3, which yields *b* = *ki*/*g* = 2 units to the punisher. Thus, provided the reasonable assumption that cooperation pays holds (*k*>*g*), punishment remains unlikely to represent a social dilemma.

It is generally held that punishers cannot invade [4], but as shown in Equation 2, where its short term cost is outweighed by longer term benefits through increased cooperation of partners, it can be self-interested. Punishment can invade where there is sufficient chance of re-meeting the punished and for them to respond by cooperating such that punishers recoup their costs.

As stated above, the analysis thus far assumes that cooperation is below maximal so it is possible for punishment to increase it; that there are sufficient future rounds for a punished individual to respond; and that each additional act of punishment will have an additive effect on the level of cooperation. Such assumptions are reasonable for examining invasion conditions, but as punishment and cooperation spread, so the chances of the assumptions being violated will increase. We could approximate the effect of whether there are sufficient rounds for a punished individual to respond by incorporating functions dependent on the probability that interactions continue (*w* as in [1]). This would encapsulate the logic that as the chance of repeated interactions declines, so will the benefits of punishing. We could also approximate the effect of the proportion of punishers *x*, such that the condition for punishment to pay would be a function of the proportion of non-punishers. It also formalizes the prediction that as punishers become more common, so the additive effect of punishment in increasing cooperation will asymptote. However, analytical solutions become unwieldy, especially as the switch from defecting to cooperating involves a step change. Rather than attempt to numerically predict when punishment will pay in relation to varying numbers of rounds and frequencies of punishers, I leave the prediction of the evolutionary dynamics to the simulations below and instead move on from analysing the simplified invasion conditions above to determining whether we can expect punishment to be stable.

To consider stability of punishment, I examine the case where increasing punishment would have no effect and one therefore prefers others to do the punishing. Table 1b considers the case where punishing has no additional effect given that one other member of the group is already punishing a responsive defector. This scenario may arise where one act of punishment is so effective that the responsive defector cannot respond by increasing its cooperation any further even if it were to be subjected to more punishment. Non-punishers then do best when another group member punishes the responsive defector (*s*>*s*-*p*) but if no other individual is punishing then it can be best to punish provided *s*>*p*. Such a payoff matrix resembles a chicken, snowdrift [51] or producer-scrounger game [52]. In such a game we can expect a mixture of punishing and non-punishing strategies to persist at equilibrium due to the diminishing returns of punishment as it increases in frequency (cf. [3] but note that these authors reported any other outcome was also possible in their models).

We can show that there will be an equilibrium at an intermediate frequency *x* of punishers by solving for when the fitness of punishers *W_P_* equals that of non-punishers *W_N_*. Assuming that punishers pay the cost of punishing; that, as discussed in the previous paragraph, punishment is non-additive in its effects, such that the presence of other punishers would not increase cooperation further; that the public goods game is repeated for sufficient rounds for punishers to get the full benefits of increased cooperation; and with a baseline fitness of 1; then *W_P_* = 1−*p*+*qb*/*c*. Non-punishers meeting a punisher get the benefits of increased cooperation in the group caused by the punisher's act without paying the costs of punishing; whereas on their own they just get the baseline fitness, so: *W_N_* = *x*(1+*qb*/*c*)+(1-*x*). Thus, an intermediate equilibrium will occur at *x* = 1-*p*/(*qb*/*c*). Taking values from [20] of *p* = 1, *q* = 3, *b* = 0.5, *c* = 0.5, this would suggest an approximate equilibrium frequency of punishers in this case of *x* = 2/3.

We can therefore see that there will be a range of payoff matrices generated by the punishment and cooperation games and that attempting to view them within any one framework, be it as a public goods game or a snowdrift game will be too limited. Rather, it is better to identify the fitness functions of different strategies in relation to their cost and benefit functions and strategy frequencies [53]. Such an approach shows how varying the parameters readily leads to transitions between recognized game matrices. In order to test how the insights gained above apply to evolving levels of cooperation and punishment strategies, I turn to using simulation techniques.

## Simulation of how cooperation and punishment evolve

The previous section addresses the question of when a co-operator should punish a responsive defector. Here I consider the evolution of cooperation and punishment strategies. A simulation model was developed in which cooperation and punishment strategies were genetically determined. Unlike in most previous models, defectors were, as in the analytical section above, responsive to punishment. Thus those that experienced punishment could reduce their losses by cooperating, meaning that punishers could manipulate responsive defectors into cooperating with them during the course of an interaction. Such a responsive strategy is analogous to the way in which Tit-for-Tat [1] responds to experience. Agents had a cooperative strategy of either cooperator *C* or responsive defector *D*. If agents cooperated, they paid a cost *i* which resulted in a group benefit *ki* where *k* was a multiple applied to cooperative investments in the group. Cost *i* was a fixed quantity within simulations but was varied between simulations; thus cooperating was a binary decision and there was no continuous variation in generosity between individuals. Each individual in a group of size *g* then received *ki/g* for each cooperative act, giving a net cost of *i*-*ki*/*g*. All individuals also had a punishment strategy of punish *P* or non-punishing *N*. Punishment involved paying a cost *p* to impose a cost *q* on a punished individual. Again, costs *p* and *q* were fixed within simulations and varied between simulations, thus the decision to punish was a binary one. Punishment could only be inflicted on defectors – I therefore did not consider antisocial punishment [24]. All combinations *DN DP CN CP* were considered as separate strategies so that strategies could not do well due to linkage [33]. Simulations were initialized with DN at 100% so C and P could only arise through mutation and invasion.

Defectors responded to the punishment regime experienced in the following way. When the sum of the costs experienced by defectors was greater as a result of being punished than as a result of cooperating, they switched behaviour to cooperation, and vice-versa. Specifically, responsive defectors cooperated when the sum of the costs they had incurred through being punished exceeded the net costs they had incurred cooperating (i.e. their investment in cooperation minus the benefit which they, like all individuals in the group, received from this) and switched back when the reverse was true. Thus punishers could manipulate responsive defectors into cooperating with them within their own lifetimes, while those that experienced punishment could reduce their losses by cooperating. However, once responsive defectors had expended more on cooperating than they had lost being punished they switched back to defecting.

The model was based on a meta-population or island structure (in order to avoid effects of genetic drift that can occur when considering a single population [54]) in which each of 10 islands contained a population of *N* = 3000 agents. Unless otherwise stated, agents began with an endowment of 6 points in order to avoid complications from negative fitnesses. Interactions were played out within fixed groups of size *g* randomly chosen from the population. Within groups, agents were selected in turn and given an opportunity to cooperate according to their strategy, as described above. Once all had played within the group, each was then given the opportunity to punish any agents who had defected (i.e. for every pairwise combination of agents, each agent with strategy *P* punished the other agent, if and only if the latter had defected, by paying a cost *p* to inflict cost *q*). These procedures were repeated for *m* meetings (where *m* = 20 unless otherwise stated) within each of the *N*/*g* groups within each island. At the end of each generation, offspring were produced for the next generation in direct proportion to the sums of the payoffs for each of the four strategy combinations. These relative payoffs were calculated both within islands, to produce the local reproductive success and across all islands to find the global reproductive success. An individual for the next generation was then derived locally with probability 0.8 and globally with probability 0.2. This method reduces the potential for genetic drift and allows migration of successful strategies among islands [54]. Note that while a responsive defector meeting a punisher may play cooperative moves during its lifetime, its payoffs nevertheless contribute to the number of responsive defectors there will be in the next generation and not to the number that inherit the cooperate strategy. In other words, it is the response rule rather than the response which is inherited. Reproduction was accompanied by mutation such that with probability μ = 0.01 an individual's strategy was replaced at random with any one of the other strategies (so, for example, adopting punishment did not first require a transition to being cooperative).

Analysis focussed on groups of 3 as these are the simplest case where cooperation cannot be explained by reciprocity. I first checked that cooperation cannot spread in the absence of punishment. With parameters *g* = 3, *k* = 2 the mean level of cooperation *C* was 0.87% (±0.002 s.e.; unless otherwise stated, all summary statistics are means across 10 simulations for generations 1000–2000). Thus cooperation did not spread above a low level. Using the same parameters but introducing punishment strategies without any dynamic adjustment of defectors to their level of punishment experienced (i.e. unresponsive defectors) gave a similar level of cooperation, *C* = 1.25% (±0.01) while punishment *P* remained at a very low level: *P* = 0.10 (±0.001). Thus punishment alone did not facilitate cooperation. This is consistent with previous models where punishment cannot invade [6], [27].

**Figure 1:**
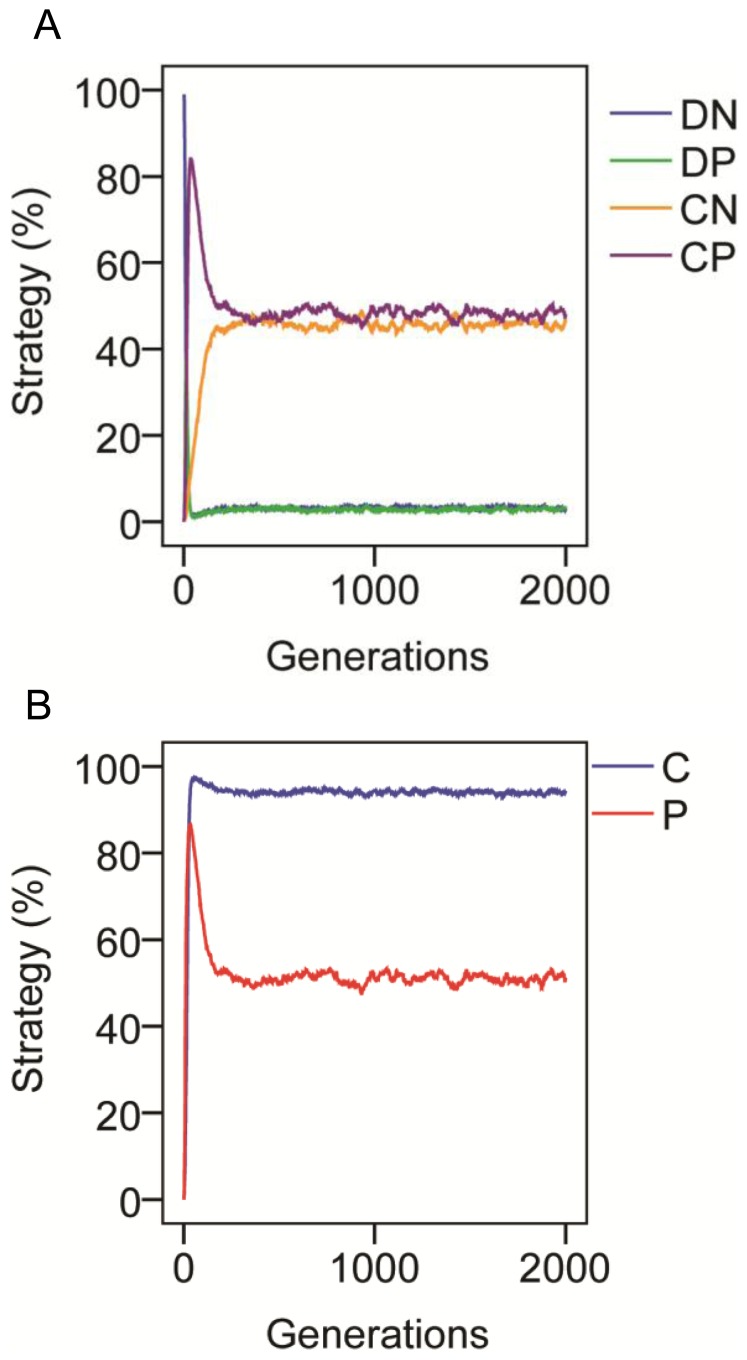
The evolutionary dynamics of cooperation and punishment. Representative example of strategy dynamics from the first of 10 simulations with parameters *g* = 3, *k* = 2, *p* = 1, *q* = 3, showing (a) frequencies of all strategy combinations (C = cooperator, D = responsive defector; P = punisher, N = non-punisher) and (b) summed frequencies of cooperative and punishing strategies.

I then introduced the facultative adjustment rule described above whereby Defectors responded to being punished. With typical economic experiment values *g* = 3, *k* = 2, *p* = 1, *q* = 3, both punishing and cooperative strategies invade from zero levels and come into equilibrium (Fig. 1). The mean strategy percentages across 10 simulations were *DN* = 3.23 (±0.009), *DP* = 2.83 (±0.02), *CN* = 45.91 (±0.08), *CP* = 48.03 (±0.10) (Fig. 1a). This resulted in an overall mean cooperation level of 93.94±0.02 and mean punishment of 50.86 (±0.09) (Fig. 1b). Note that although only the first 2000 generations are given, levels were stable for much longer e.g. means across 10 simulations between generations 9000 and 10000 were: *C* = 93.98 (±0.03), *P* = 50.93 (±0.08).

Above, it was predicted that punishment can pay where *p*<*qb*/*c*. To test how punishment and cooperation varied with their costs and benefits, the ratios *p*∶*q* and b∶c were varied (the latter through varying *k*∶*g*). As predicted, a high level of cooperation was found only where the equation above held true (Table 2).

**Table 2:** The leveraging effect of cost to benefit ratios on cooperation and punishment.

			*p/q*	
		1/1	1/2	1/3
	1/3	0.09±0.008 0.26±0.001	0.19±0.009 1.48±0.007	96.39±0.04 44.71±0.11
*k/g*	1.5/3	1.03±0.002 19.55±0.03	92.56±0.04 48.49±0.06	95.17±0.03 41.98±0.09
	2/3	80.81±0.10 45.95±0.04	92.64±0.03 48.36±0.08	93.94±0.02 50.86±0.09

Varying *k* with *g* = 3 and varying *q* with *p* = 1. Cells give mean (± s.e.m.) cooperation (first figure) and punishment (second figure), each computed for generations 1001–2000 and averaged across 10 simulations. The area below the stepped line represents the condition *p*<*qb*/*c* (where *c* = *i* -*ki*/*g* and *b* = *ki*/*g*).

As shown above, cooperation and punishment can only establish in this system with the response rule. Therefore it is not surprising that with average number of meetings *m* = 1, there is little of either (C = 11.84±0.04, P = 1.89±0.01). However 4 meetings are sufficient for levels of cooperation and punishment almost identical to those for the standard conditions of *m* = 20 (C = 93.50±0.04, P = 32.25±0.10) while adding further meetings has little effect (e.g. with *m* = 30, C = 96.54±0.02, P = 38.20±0.12).

While punishment facilitated cooperation, amongst cooperators there tended to be a high proportion of non-punishers (Fig. 1). In groups of 3, if one punishes a responsive defector then it is only beneficial for another also to punish the responsive defector if there is an additive effect on resulting cooperation. If the parameters are changed so that there is an additive effect of 2 individuals in a group of 3 both punishing then higher levels of punishment result (e.g. with *g* = 3, *k* = 2, *p* = 0.2, *q* = 0.3, *P* = 70.69±0.03 and *C* = 63.03±0.12). Note that here cooperation is lower due to the less favourable *p*∶*q* ratio.

How does group size affect cooperation and punishment? Substituting into *p*<*qb*/*c* where *b* = *ki*/*g* and *c* = 1-*ki*/*g* and rearranging, we get
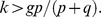
This formula is of value in deducing how great the synergistic effects of cooperation must be for punishment to invade in groups of different sizes. For example, with *g* = 3, *p* = 1, *q* = 3, *k* must be >0.75; with *g* = 8 *k* must be >2; and with *g* = 20, *k* must be >5. These analytical results were confirmed by simulation.

## Discussion

These results demonstrate that punishment can facilitate the invasion of cooperation in repeated games which then persists in stable equilibrium at a high level. Thus, in contrast to previous models, punishment can both invade and resist invasion. There is no requirement for a critical frequency of punishers and the dynamics do not result in alternative equilibria [3], [27]. Because punishment is costly it has been almost universally assumed that it constitutes a social dilemma [4]–[11], [20], [55], [56]. However, what has typically been overlooked is that this need not be the case provided a punisher's own benefit from punishing exceeds its costs. This becomes increasingly likely if we allow responsive strategies such that punished individuals switch to cooperating. It has previously been suggested that individuals may cooperate more with those that are more likely to punish them [6]; this paper demonstrates one mechanism as to how that may be achieved. The mechanism of switching to cooperation after being punished is not only highly plausible but has been demonstrated in natural systems [16].

A recent study found evidence for punishment being carried out for self-serving reasons: if a female cleaner wrasse cheats on a client, then a male servicing the same client may punish this female [46]. This encourages the female to be more cooperative, which in turn benefits the male as he is less likely to lose a client. Interestingly, punishment in this instance may be facilitated by the greater strength of males, which fits with the analysis showing that punishment is more likely to be found where the ratio of costs favours the punisher.

Applying this to recent experimental results, it can be understood why people might employ punishment in public goods games. In typical experiments the relation *p*<*qb*/*c* will hold; e.g. in [20], *g* = 3, *k* = 1.5 (so *b* = 0.5 and *c* = 0.5), *p* = 1 and *q* = 3 therefore 1<3*0.5/0.5 holds true and punishment can be self-interested in the context of a invasion in a repeated game. The situation is somewhat more complex where cooperation is not a binary decision but a variable between 0–20 and investment in punishment varies between 0–10 points [18], [20]. Nevertheless, in [20] a small investment in punishment produces a greater increase in cooperative rewards: individuals spend on average 0.46 on punishment which raises contributions from 7.2 without punishment to 16.8 with; this in turn raises net income from 23.6 without punishment to 26.6 with punishment. It is therefore in an individual's own direct self interest to punish, at least at the average level. As shown here, it will typically take several rounds of cooperation by a punished individual (depending on the response rule) before punishing becomes profitable. Thus, the results in [20] whereby punishment becomes profitable with 50 rounds but not with only 10 rounds can be understood. Note the crucial difference that the result in [20] is interpreted in terms of group selection of individually costly behaviour whereas here I have shown it is not individually costly but individually beneficial.

Further experiments systematically varying the costs and benefits of punishing and cooperating would be valuable to test the models presented here. One study that varied punishment parameters found results consistent with the analysis presented here [57]. In their experiments, *c* = 0.5 and *b* = 0.5 and four punishment conditions were employed: *p* = 1, *q* = 3; *p* = 1, *q* = 1; *p* = 3, q = 3; *p* = 3, *q* = 1. Substituting into p<qb/c we find that only the *p* = 1, *q* = 3 case satisfies the condition for punishment to pay. This is consistent with the experimental findings; cooperation increased only in this condition. The significance of this is that individuals are sensitive to the costs and benefits they receive and are not simply punishing out of altruistic or other-regarding motivations as has been proposed.

An interesting aspect of the simulation results is that in groups of three or more, punishers and non-punishers come into stable equilibrium. This is because punishment is only worthwhile if it provides an additive benefit; if not then it may be better to allow another individual to do the punishing. In such circumstances, the scenario becomes a snowdrift game [51] whereby the best response to cooperation is defection and vice versa, as discussed at the end of Section 2. A ‘producer-scrounger’ type of equilibrium is established between punishers and non-punishers. As demonstrated by [53], where individuals invest in a resource (in this case punishment) from which all in a group benefit, the payoffs experienced can shift through mutual benefit to snowdrift to public goods game as parameters are varied.

The current findings compare with those of [58] who showed that cooperation could be supported if players know the reputations of their co-players, i.e. whether they have punished or not. In the current model, players do not need to know reputations, they simply respond to the level of punishment to which they have been subjected.

An empirical study of punishment in repeated games has reported that non-punishers achieve higher payoffs than punishers [21]. This was interpreted as showing that costly punishment was maladaptive, so appears at odds with the predictions made here. However, the study of [21], as well as theoretical work by [45], [59], uses a scenario which differs fundamentally from the rest of the literature on punishment. In these studies, punishment is an alternative to cooperation rather than being a subsequent decision. Furthermore, they consider only pair-wise interactions in which cooperation is not problematic because it is well established that full cooperation can be readily achieved through strategies such as Tit-for-Tat [1]. The question these authors consider is effectively whether a population of individuals playing Tit-for-Tat-based full cooperation can be invaded by individuals which will reduce their own payoff to reduce their partner's payoff. By definition this cannot result in increased payoffs, so the conclusion that “winners don't punish” simply recovers the particular assumptions behind these specific studies and has no general meaning. Indeed, the issue of punishment does not arise in pairs: it is only in larger groups that punishment may be of value because it offers a means to direct retaliation at individuals whereas defection effectively punishes all group members whether they have cooperated or not [3].

The dilemma of a public goods game is that an individual's stake in the cooperative benefits is less than their investment, but in the punishment game, each point spent on punishment can yield more points in terms of increased cooperation. Therefore the public goods game of cooperation can be solved by superimposing a self-interest game. The significance of this is that punishment has been cited as one of the main planks of evidence for a proposed phenomenon of “strong reciprocity” in which individuals are said to reward altruists and punish defectors even when this runs contrary to self interest [60]. Advocates of this view have claimed that such behaviour challenges the self-interest paradigm [26] that has been fundamental to economic and evolutionary approaches. They have gone on to claim that other-regarding preferences are the consequence of culturally group-selected norms [61].

Models of punishment in the context of strong reciprocity have claimed to demonstrate how punishment can spread [60], [62]. However, this result is subject to the unrealistic constraint that cooperating and punishing must be tightly linked [33]. The interpretation is further clouded by the modellers' confusion over what is driving selection in these systems: Lehmann et al conclude that punishment actually spreads because helping functions as a tag allowing spiteful behaviour towards non-punishers.

If, as I have demonstrated, punishment is typically not a social dilemma then it cannot provide evidence of group-selected other-regarding preferences, at least in the repeated games studied here and by e.g. [20]. Cooperating and punishing in one-shot interactions will remain a subject of debate, but just as reciprocity in repeated games [1] helps elucidate cooperation in one-shot games [41] so understanding when punishing can pay in repeated games should lead us toward understanding why people continue to punish in one-shot interactions. Explanations invoking group selection [27], opt-outs [10], reputation benefits [19], [22], coordination [12] and pool punishment [8] may therefore extend the range of parameters in which punishment pays, including to larger group sizes than considered here, but such mechanisms may not be necessary to explain punishment in small groups of repeatedly interacting individuals. The argument that punishment can be self-interested has been applied to recent work on animals [46], [63]. Punishment can pay in humans too, and may therefore be an important force stabilizing group cooperation.
